# Small cell lung cancer stem cells display mesenchymal properties and exploit immune checkpoint pathways in activated cytotoxic *T* lymphocytes

**DOI:** 10.1007/s00262-021-02998-1

**Published:** 2021-07-06

**Authors:** M. Alper Kursunel, Ekim Z. Taskiran, Ece Tavukcuoglu, Hamdullah Yanik, Funda Demirag, Beren Karaosmanoglu, Feyza Gul Ozbay, Aysegul Uner, Dorina Esendagli, Derya Kizilgoz, Ulku Yilmaz, Gunes Esendagli

**Affiliations:** 1 Department of Basic Oncology, Hacettepe University Cancer Institute, 06100 Sihhiye, Ankara, Turkey; 2 Max-Delbrück-Center for Molecular Medicine, Robert-Rossle Str. 10, 13125 Berlin, Germany; 3 Department of Medical Genetics, Faculty of Medicine, Hacettepe University, Ankara, Turkey; 4 Department of Pathology, Atatürk Chest Diseases and Thoracic Surgery Training and Research Hospital, Ankara, Turkey; 5 Department of Pathology, Faculty of Medicine, Hacettepe University, Ankara, Turkey; 6 Department of Chest Diseases, Faculty of Medicine, Baskent University, Ankara, Turkey; 7 Department of Chest Diseases, Atatürk Chest Diseases and Thoracic Surgery Training and Research Hospital, Ankara, Turkey

**Keywords:** Lung cancer, Cancer stem cell, Immunotherapy, PD-1, TIM-3, LAG3

## Abstract

Small cell lung cancer (SCLC) is an aggressive tumor type with early dissemination and distant metastasis capacity. Even though optimal chemotherapy responses are observed initially in many patients, therapy resistance is almost inevitable. Accordingly, SCLC has been regarded as an archetype for cancer stem cell (CSC) dynamics. To determine the immune-modulatory influence of CSC in SCLC, this study focused on the characterization of CD44^+^CD90^+^ CSC-like subpopulations in SCLC. These cells displayed mesenchymal properties, differentiated into different lineages and further contributed to CD8^+^ cytotoxic T lymphocytes (CTL) responses. The interaction between CD44^+^CD90^+^ CSC-like cells and T cells led to the upregulation of checkpoint molecules PD-1, CTLA-4, TIM-3, and LAG3. In the patient-derived lymph nodes, CD44^+^ SCLC metastases were also observed with T cells expressing PD-1, TIM-3, or LAG3. Proliferation and IFN-γ expression capacity of TIM-3 and LAG3 co-expressing CTLs are adversely affected over long-time co-culture with CD44^+^CD90^+^ CSC-like cells. Moreover, especially through IFN-γ secreted by the T cells, the CSC-like SCLC cells highly expressed PD-L1 and PD-L2. Upon a second encounter with immune-experienced, IFN-γ-stimulated CSC-like SCLC cells, both cytotoxic and proliferation capacities of T cells were hampered. In conclusion, our data provide evidence for the superior potential of the SCLC cells with stem-like and mesenchymal properties to gain immune regulatory capacities and cope with cytotoxic T cell responses. With their high metastatic and immune-modulatory assets, the CSC subpopulation in SCLC may serve as a preferential target for checkpoint blockade immunotherapy .

**Supplementary Information:**

The online version contains supplementary material available at 10.1007/s00262-021-02998-1.

## Introduction

Small cell lung cancer (SCLC) is a neuroendocrine malignancy which constitutes approximately 13% of lung cancers worldwide [1–3]. The patients commonly benefit from first-line chemotherapy; however, recurrence occurs very early in most SCLC cases [4, 5]. The development of chemoresistance subsequent to disease progression and the distant metastases critically limit overall survival [6]. Theoretically, chemotherapy-resistant cancer stem cells (CSCs), which constitute a very minor subpopulation, are responsible for drug resistance and aggressiveness in SCLC [7–9]. CSCs are frequently identified with mesenchymal markers such as CD44, CD90, CD87, and CD133 [9–11]. Accordingly, with its multipotent neuroendocrine origin and superior dissemination capacity, SCLC has been acknowledged as an archetype for CSC research [9]. Nevertheless, even though the immune regulatory capacities of CSCs are well-acknowledged, their influence on the anti-tumor immunity in SCLC remains to be better elucidated.

Stem cells (SCs), undifferentiated cells with self-renewal capacity, are responsible for regeneration and located in stem cell niches to maintain their characters and numbers [12]. CSCs similar to tissue SCs are programmed to protect themselves from external threats by commonly expressing ABC transporters against cytostatic/cytotoxic drugs and downregulation of MHC class I or NKG2D ligands to protect themselves against anti-tumor reactions [13]. In order to modulate local immune responses, CSCs have the capacity to secrete anti-inflammatory soluble factors such as IL-10, IL-13, Galectin-3, GDF-15, Transforming growth factor (TGF)- β 1/2, prostaglandin E2 (PGE2) [14, 15]. In further detail, CSCs regulate monocyte differentiation by producing macrophage inhibitor cytokine 1 (MIC1) which in turn drive differentiation of monocytes toward the tumor-promoting type of macrophages or by expressing CD200 to inhibit macrophage activation together with CD47 to block phagocytosis [15–18]. Furthermore, CXCL12 secreted by CSCs recruit regulatory myeloid cells into the tumor microenvironment consisting of MDSCs, suppressive dendritic cells, and Tregs [14]. Expression of IDO from CSCs leads to inhibition of *T* cell proliferation and skew Th2 type immune responses which are more favorable for TME than destructive Th1 type [19]. In addition to common PD-L1 expression, CSCs from some cancer types were shown to express Galectin-3, a ligand of LAG3, Galectin-9, a ligand of TIM-3, and even the expression of CTLA-4 and TIM-3 receptors indicating a close relationship between CSCs and immune checkpoint molecules [20–22]. More interestingly, CD44^+^ stem cells emerged adaptive immune resistance by engaging CTLA-4 receptor on *T* cells upon acquiring CD80 expression [23].

In response to inflammatory mediators such as interferon (IFN)-γ, which is the primary mediator of *T* cell-mediated anti-tumor immunity, cancer cells initiate an adaptive (secondary) resistance program to survive anti-tumor immune responses [24, 25]. In this regard, they upregulate regulatory ligands which directly suppress the activated *T* cells through co-inhibitory receptors such as programmed death-1 (PD-1), cytotoxic *T*-lymphocyte associated protein 4 (CTLA-4), *T*-cell immunoglobulin mucin-3 (TIM-3) and lymphocyte-activation gene 3 (LAG3) [24, 26]. Moreover, high surface levels of inhibitory receptors indicate a hyporesponsive state where the cytotoxicity against cancer cells is impaired [27]. Accordingly, blockade of the interaction between PD-1 and its ligands, PD-L1 and PD-L2, is one of the most successful cancer immunotherapy modalities, especially in melanoma, renal cell cancer, and non-small cell lung cancer [28]. Nevertheless, the checkpoint blockade therapies are essentially successful toward immunogenic tumors which are highly infiltrated by CD8^+^ cytotoxic T lymphocytes (CTL) [29]. SCLC has been regarded as an immunogenic malignancy since it displays a high mutation burden and bears many neoantigens [30, 31]. In addition, autoimmune paraneoplastic syndromes are common in SCLC patients [31]. Even though immune infiltration is limited in primary SCLC tumors, in metastatic lymph nodes, the interaction between SCLC cells and T cells is almost inevitable. Nevertheless, clinical trials reported a limited efficacy for targeting the PD-1/PD-L1 axis in SCLC patients [32].

This study aims to characterize the CSC-like subpopulations within SCLC cells and to determine their influence on CTL responses. Here, CD44^+^CD90^+^ cells in SCLC displayed mesenchymal and stemness properties which facilitate lymph node metastasis. Intriguingly, these CSC-like subpopulations maintained immune responses which lead to the upregulation of multiple inhibitory receptors on CD8^+^ T cells. Moreover, they acquired immune-modulatory capacities, partly through PD-L1 and PD-L2 molecules, upon exposure to immune reactions and IFN-γ.

## Materials and methods

### Cell culture and isolation of SCLC subpopulations

SCLC-21H, NCI-H82 (DSMZ), and NCI-H69 (ATCC, LGC Promochem) SCLC cell lines were maintained as suspension cultures in complete H-glucose DMEM (SCLC-21H) or RPMI 1640 medium containing 20% fetal bovine serum (FBS), penicillin (100 U/mL), and streptomycin (100 µg/mL) (Lonza) at 37 °C under a 5% CO_2_ atmosphere. The subpopulations of NCI-H82 and NCI-H69 (H82Adh and H69Adh, respectively) were established by long-term serial passaging of the cells that adhered onto the culture flask. H69Sc cells were isolated from the H69Adh subpopulation through CD44 positive selection by magnetic-activated cell sorting (MACS) (Miltenyi) which was followed by fluorescence-activated cell sorting (FACS) of CD44^+^CD90^+^ cells. For certain experiments, SCLC cells were treated with 150 ng/mL recombinant IFN-γ (R&D Biosystems) for 24 or 48 h. Peripheral blood mononuclear cells (PBMC) were isolated from healthy donors’ blood samples (Hacettepe University Local Ethics Committee, approval no. GO 17/503) by density gradient centrifugation (Ficoll 1.077; GE Healthcare). PBMC cultures were maintained in complete RPMI 1640 medium with 10% FBS.

### Flow cytometry and FACS

The monoclonal antibodies (mAb) anti-human-CD44 (BJ18), -CTLA-4 (L3D10), -LAG3 (11C3C65), -PD-1 (EH12.2H7), -PD-L2 (24F.10C12), -TIM-3 (F38-2E2) (BioLegend, USA); CD90 (5E10) (Thermo Fisher Scientific); CD4 (SK3), CD8 (SK1), CD25 (BC96) (Sony Biotechnology); CD107a (H4A3), CD137 (4B4-1), CD69 (FN50) (BD Biosciences) were used in immunophenotyping analyses and FACS. The percentage of positive cells was determined in comparison with the isotype-matched antibody controls. For cell isolation and purification, PBMC were labeled with anti-CD8 and -CD56 antibodies, and cytotoxic T cells were sorted as CD56^−^CD8^+^ lymphocytes with a purity of > 98%. For different assays, CD8^+^ T cells were purified from the co-cultures established with SCLC cells according to their TIM-3 and LAG3 expression (TIM-3^+^LAG3^+^ and TIM-3^−^LAG3^−^ populations). Flow cytometric immunophenotyping and cell sorting were performed on a FACSAria II (BD).

## Cell differentiation assays

Adipocyte, osteocyte, and chondrocyte differentiation capacities of SCLC cells were tested with commercial differentiation kits (StemPro, Thermo Fisher Scientific) in accordance with the manufacturer’s recommendations. Following the 21-day culture in differentiation media, which were refreshed every 3 days, the cells were fixed with 4% paraformaldehyde and stained with Oil Red O, Alizarin red, and methylene blue which designate adipogenic, osteogenic, and chondrogenic differentiation, respectively.

## Migration assay

SCLC cells (2 × 10^5^/100 µL) were resuspended in 1% FBS-containing RPMI 1640 medium and were seeded into the upper chamber of the transwell with 8 µm pore polycarbonate membrane insert (Corning), whereas the bottom chamber was filled with 10% FBS-containing culture medium. After 16 h of incubation at 37 °C with 5% CO_2_, the cells found in the lower chamber and the cells adhered to the bottom of the membrane were stained with Giemsa and counted under a light microscope.

## Polarization analysis

SCLC cells were incubated for 16 h on chamber slides (BD) coated with fibronectin (Sigma-Aldrich) and, then, fixed in 4% paraformaldehyde and permeabilized with 0.1% Triton X-100 (Sigma-Aldrich) in PBS, blocked with 10% BSA (Sigma-Aldrich) for one hour. Following the staining with phalloidin-iFluor 488 reagent and DAPI (Abcam), the slides were mounted; micrographs were taken under a fluorescent microscope and processed by ImageJ software (NIH).

## Transcriptomic analyses with next generation sequencing (NGS)

Total RNA was isolated (RNeasy, QIAGEN) from human pulmonary alveolar epithelial cells (PAEpiC) type II cells (Sciencell), SCLC-21H, NCI-H82, NCI-H69, H82Adh, H69Adh, H69Sc, mesenchymal stem cells (MSC) (ATCC, LGC Promochem) or from the SCLC cells after 24 h of IFN-γ treatment. Then, barcoded cDNA libraries were generated from 10 ng of total RNA by VILO Superscript cDNA synthesis kit (Thermo Fisher Scientific). Ultra-high multiplex PCR was performed with Ion AmpliSeq™ Human Gene Expression Chef Ready Kit (Thermo Fisher Scientific). The libraries were obtained (Ion Chef, Thermo Fisher Scientific) and clonally amplified by emulsion PCR (Ion PI Hi-Q OT2 200 Kit, Thermo Fisher Scientific) on an Ion Torrent OneTouch2 instrument. The templated libraries were sequenced on an Ion Proton semiconductor sequencing system. The data were processed by Torrent Suite analysis pipeline and raw reads were mapped to the human genome assembly hg19 AmpliSeq Transcriptome version by Torrent mapping alignment software and normalized according to the "read per millions" method. Differentially expressed genes (DEG) were determined with DESeq2, the changes more than eightfold were considered as significant. The genes were clustered according to the Kyoto encyclopedia of genes and genomes (KEGG) pathway enrichment analyses or Gene Ontology (GO) pathways that defines functional characteristics of the samples.

## Co-cultures

Co-cultures were established with the SCLC cells (2.5 × 10^4^) and PBMC at a 1:4 ratio in the presence of anti-CD3 mAb (25 ng/mL, HIT3a) in U-bottom 96-well plates. At different time points of incubation, either the SCLC cells were detached by accutase treatment (BioLegend) or the PBMC found in suspension were collected for further analyses. To test the effect of soluble factors, conditioned media are collected after 48 h of culture and used in co-cultures at a 1:1 ratio. The SCLC cells pretreated with IFN-γ were also used in the co-cultures or IFN-γ neutralizing mAb (5 μg/mL, B27) or isotype-matched control IgG (5 μg/mL) were added to certain experimental setups.

## Flow cytometric proliferation analysis

PBMC or CD8^+^ T cells purified according to TIM-3 and LAG3 expression were resuspended in serum-free RPMI 1640 (5 × 10^6^ cells/mL) and stained with 5 μM eFluor670 (eBioscience) or carboxyfluorescein succinimidyl ester (CFSE, Invitrogen). Following several washing steps with complete medium, PBMCs were used in co-cultures. Following 96 h, CD8^+^ *T* cells were gated and their proliferation was assessed by flow cytometry according to eFluor670 dilution. Alternatively, the purified CD8^+^ cells were further stimulated with anti-CD3/CD28 beads (1:1 bead-to-cell ratio, Dynabeads, Thermo Fisher Scientific) or with anti-CD28 (2 µg/mL) in an anti-CD3 (2 µg/mL) coated plate for 72 h.

## Multi-analyte ELISA array

Interleukin (IL)-2, IL-4, IL-6, IL-10, TNF-α, IFN-γ, perforin, sFASL, granzyme A, and granzyme B concentration secreted into the PBMC:SCLC co-cultures was determined from the supernatants collected at 96 h by a flow cytometry-based enzyme-linked immunosorbent assay (ELISA) array (LEGENDplex™ Human CD8/NK Panel, BioLegend). The amount of cytokines was analyzed according to mean fluorescence intensity (MFI) values of the samples and standards by using the data analysis software supplied by the manufacturer.

## Tumor formation capacity of SCLC cells

Eight-week-old CD1-nude mice (Kobay A.Ş., Turkey) were used with ethical approval from the local ethics committee (Doc. Nr.:251). Parental (5 × 10^5^, 2 × 10^6^, and 10^7^ cells/100 μL) and adherent subpopulations of SCLC cells (5 × 10^5^ and 2 × 10^6^/100 μL) were dispersed in serum-free RPMI 1640 medium and injected subcutaneously. Parental cells and their adherent subpopulations were inoculated on the right and the left sides, respectively, of the mice. The tumor growth was followed for 6 weeks.

## Immunohistochemistry

Sections (4 μm thick) from archived paraffin-embedded primary tumor and metastatic lymph node specimens of SCLC patients were taken and placed on polylysine microscope slides. Following deparaffinization, dehydration, and blocking of endogenous peroxidase activity, the tissue sections were incubated with primary antibodies, anti-CD3 (prediluted; 2GV6, Ventana), -CD44 (1/50 dilution; 156-3C11, Leica), -PD-1 (1/250 dilution; EH12.2H7, BioLegend), -TIM-3 (1/1500 dilution; OTI2E2, Origene), and -LAG3 (1/625 dilution; EPR4392, Abcam). After washing steps, incubations with appropriate secondary antibodies, streptavidin–biotin complex, horseradish peroxidase (HRP), and 3,3′-Diaminobenzidine (DAB) chromogen were performed. Immunohistochemical staining was observed and documented by conventional light microscopy.

## Statistical analysis

The results were obtained from at least three independent experiments. The data are presented as mean with standard deviation (SD) or standard error (SE). Mann–Whitney *U* test, Student’s paired or unpaired t-test, ANOVA, or Chi-square tests were used where appropriate to show the statistical differences. An associated P-value lower than 0.05 was considered to be statistically significant.

## Results

### Stem-like SCLC populations are characterized with mesenchymal properties

**Fig. 1:**
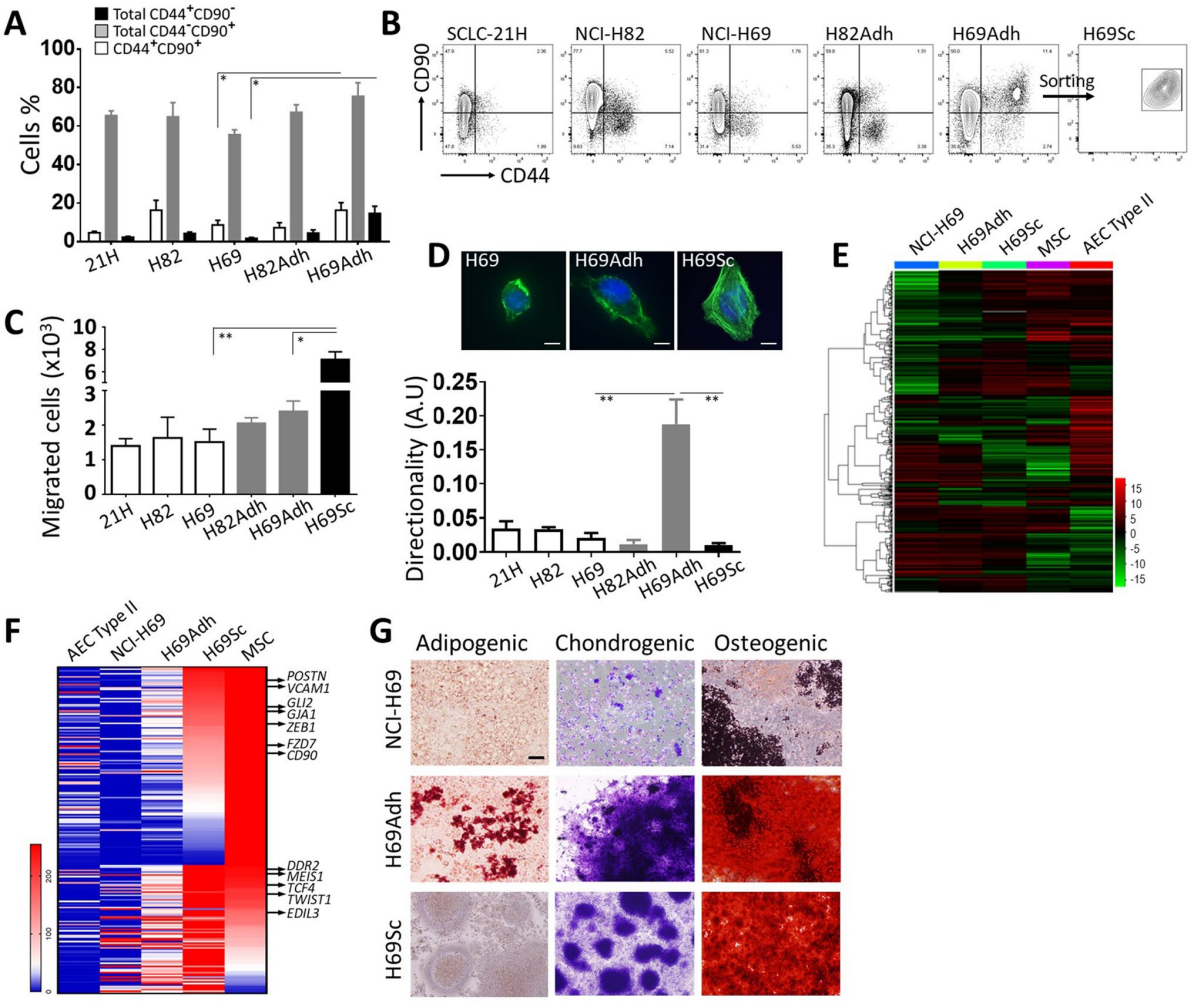
Characterization of SCLC subpopulations with stem-like and mesenchymal properties. **a** Percentage distribution and **b** representative flow cytometry plots of SCLC cell populations according to CD44 and CD90 expression are shown. **c** Migration toward high FBS gradient and **d** polarization on fibronectin substratum are given for different SCLC subpopulations. Representative micrographs for the polarization of NCI-H69, H69Adh and H69Sc cells are shown (scale, 10 µm). Heatmap analyses **e** for global transcriptomics data and **f** for the genes related to mesenchymal cells and stemness in NCI-H69 subpopulations, alveolar epithelial cell type II (AEC II), and mesenchymal stem cells (MSC). **g** Adipogenic, chondrogenic and osteogenic differentiation capacities of NCI-H69, H69Adh and H69Sc cells. Representative micrographs obtained with oil red O, methylene blue, and Alizarin red staining are demonstrated

In vitro, primary SCLC cells and established cell lines such as SCLC-21H, NCI-H82, and NCI-H69 tend to form aggregates and grow in suspension [33, 34]. The relationship between adherence and stemness has been previously reported in SCLC [33]. When grown in contact with the surface of culture flasks, adherent subpopulations of NCI-H69 and NCI-H82 cell lines emerged and maintained as monolayer cultures, H69Adh, and H82Adh (Supplementary Fig. 1). These adherent cells grew in tight clusters and gave rise to loosely adherent cells (Supplementary Fig. 1) which were viable and remained in suspension (data not shown). In accordance with the literature, both suspension and adherent SCLC cells used in our study commonly expressed CD90, whereas CD44 expression was restricted to a minor subpopulation (Fig. 1a, b). In H69Adh, a group of cells was clearly identified with CD90 and CD44 co-expression (Fig. 1a, b). These CD44^+^CD90^+^ cells were further purified as H69Sc subpopulation and maintained in vitro (Fig. 1b and Supplementary Fig. 1).

Next, the parental SCLC cell lines (SCLC-21H, NCI-H82, NCI-H69) and their derivatives (H82Adh, H69Adh, and H69Sc) were compared in terms of adhesion and migration properties, molecular signatures and mesenchymal stem-like features. There was no significant difference between the migratory capacity of parental cell lines grown in suspension or their adherent subpopulations; however, H69Sc cells displayed a significantly higher (~ 4.5 fold) number of migrated cells than its parental NCI-H69 and H69Adh cells (Fig. 1c). When in contact with the fibronectin-coated surface as a common component of the extracellular matrix, adherent populations gained greater surface area than suspension cells (A.U., SCLC-21H, 2061 ± 687; NCI-H82 4917.1 ± 1639; NCI-H69, 2380 ± 660.1; H82Adh, 8199.4 ± 2472.2; H69Adh, 15,584 ± 5896.2; H69Sc, 15,469.9 ± 4896.4). Even though both H69Adh and H69Sc covered similar surface area and displayed polarized assembly of actin filaments, H69Adh was identified with explicit directionality (Fig. 1d).

Compared to primary type II alveolar epithelial cells (AEC), the transcriptome data obtained from the SCLC cells represented a significantly altered gene expression profile with a high variance ratio indicating the distinctions among the cell lines and their derivatives (Supplementary Fig. 2). Among NCI-H69, H69Adh, and H69Sc high numbers of differentially expressed genes (DEGs) were determined (> eightfold DEGs, NCI-H69 vs. H69Adh, 2436; NCI-H69 vs. H69Sc, 4397; H69Adh vs. H69Sc, 2359) which coincide with approximately 15–25% shift in respective transcriptomes (Supplementary Fig. 3A). In order to better define the mesenchymal, epithelial and stem cell-like features of NCI-H69 and its derivatives, their transcriptomic data were compared with that of MSC and type II AEC. Especially, H69Sc characterized by a molecular signature harboring both MSC and epithelial assets (Fig. 1e and Supplementary Fig. 3B). Expression of the genes associated with pluripotency, epithelial-to-mesenchymal transition, and migratory processes (*POSTN, VCAM1, GLI2, GJA1, ZEB1, FZD7, CD90, DDR2, MEIS1, TCF4, TWIST1, EDIL3*) were commonly and highly upregulated in H69Sc, MSC and in H69Adh to some extent (Fig. 1f). Moreover, the differentiation capacity of H69Adh and H69Sc cells into osteocyte and chondrocyte lineages confirmed their stem-like properties. Adipogenic differentiation could only be induced in H69Adh to a limited extend (Fig. 1g). Similar results were also obtained with H82Adh cells. All parental SCLC cell lines as well as NCI-H69 were reluctant to differentiate (data not shown and Fig. 1g). Moreover, a pilot in vivo experiment demonstrated the tumor propagation capacity of H69Adh cells at lower numbers than the parental NCI-H69 cell line (Supplementary Fig. 4).

Both mesenchymal and stem-like assets of tumor cells have been associated with increased metastatic potential in many cancers besides SCLC [35, 36]. In accordance with the literature, we showed that SCLC cells which metastasize into the lymph nodes, but not the primary tumor tissues, were highly positive for CD44 (Supplementary Fig. 5). Collectively, adherent CD44^+^CD90^+^ cells were identified as a CSC-like subpopulation among SCLC cells. Their mesenchymal and stem-like characteristics together with cytoskeletal features and migratory capacity corresponded to the CD44^+^ tumor cells’ enrichment in the metastatic lymph nodes.

### Cytotoxic *T* lymphocyte (CTL) responses are maintained by SCLC cells

**Fig. 2:**
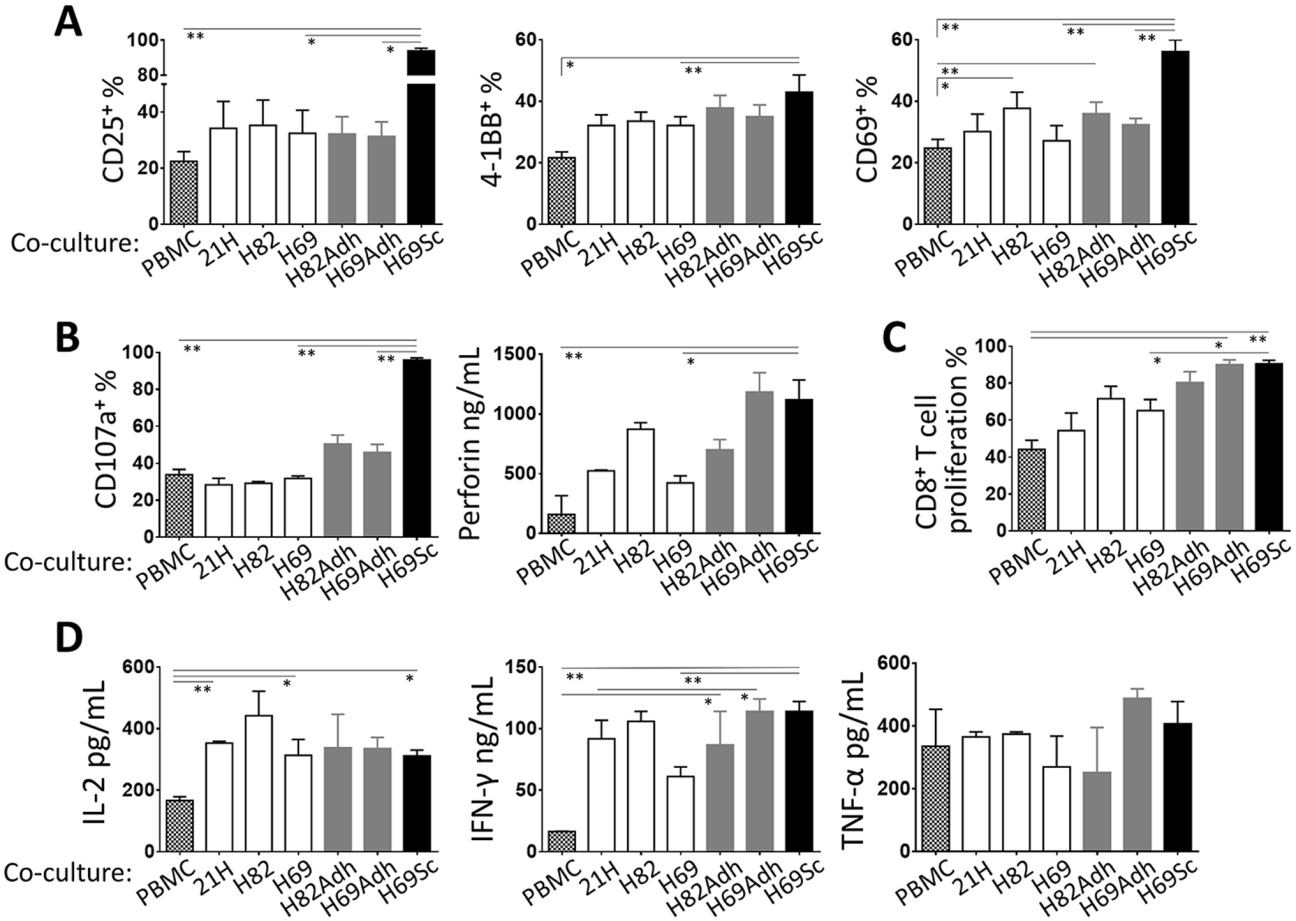
Cytotoxic T cell responses in the presence of SCLC cells. PBMCs were co-cultured with SCLC cells at 1:0.25 ratio in the presence of anti-CD3 mAb. Expression of **a** activation markers CD25, 4-1BB, and CD69 (*n* ≥ 4), and **b** cytotoxicity-related CD107a and perforin molecules upon 24 h of the co-culturing (*n* ≥ 7) was assessed by flow cytometry-based techniques. **c** Proliferation of CD8^+^ T cells and **d** secretion of IL-2, IFN-γ, and TNF-α cytokines were determined after 96 h of the co-culturing (*n* ≥ 6). (PBMC, control PBMC alone; SCLC-21H, 21H; NCI-H82, H82; NCI-H69, H69; *p < 0.05, **p < 0.01)

Our findings in SCLC tumors, which are in agreement with the literature, indicated that the CD44^+^ cells with mesenchymal and stem-like characteristics tend to metastasize to lymph nodes where they frequently encounter *T* lymphocytes [37–39]. Moreover, primary SCLC tumors are rarely infiltrated by *T* cells [40] (Supplementary Fig. 6). Therefore, we examined the influence of the SCLC cells on CTL-associated immune reactions. For this purpose, the SCLC cell lines and their derivatives were co-cultured with CD3-stimulated PBMC. In general, unlike anticipated, the presence of SCLC cells did not directly interfere with *T* cell activation. Intriguingly, certain parameters tested were even improved by the adherent populations, primarily H69Sc. Especially, the percentage of CD8^+^ *T* cells expressing the activation markers CD25, 4-1BB, and CD69 were significantly increased in co-cultures with H69Sc cells (Fig. 2a, Supplementary Fig. 7). Similarly, indicators of cytotoxic activity including secreted perforins and degranulation (CD107a^+^ %) were augmented upon co-culture with H69Sc (Fig. 2b, Supplementary Fig. 7). H82Adh and H69Adh cells positively influenced the expression of perforin and CD107a by T cells in the co-cultures. Even though CD8^+^ T cell proliferation was noticeably augmented by the adherent SCLC populations (with H82Adh, 80.37 ± 5.82%; H69Adh, 90.07 ± 2.55%; H69Sc, 90.56 ± 1.85%), induced proliferation was also found in the co-cultures with parental SCLC cell lines, especially with NCI-H69 (65.15 ± 6.05%) and NCI-H82 (71.58 ± 6.7%) (Fig. 2c, Supplementary Fig. 7). The amount of IFN-γ (PBMC only, 16.5 ng/mL; with SCLC cells, range 61–114 ng/mL) and IL-2 (PBMC only, 166.24 pg/mL; with SCLC cells, range 311.08–441.78 pg/mL), but not TNF-*α*, secreted into the co-cultures were accordingly increased with all the SCLC cell types employed (Fig. 2d). Among the parental SCLC cell lines, NCI-H82 tend to induce higher CD69 expression, perforin secretion, proliferation, and IFN-γ production by *T* cells (Fig. 2a–d).

Together, SCLC cells did not directly hamper cytotoxic *T* cell responses. Intriguingly, H69Sc and also adherent populations even simulated the *T* cell activation, proliferation, cytotoxicity, and IFN-γ secretion.

### PD-1 ligands are highly upregulated by adherent SCLC populations in response to immune responses

**Fig. 3:**
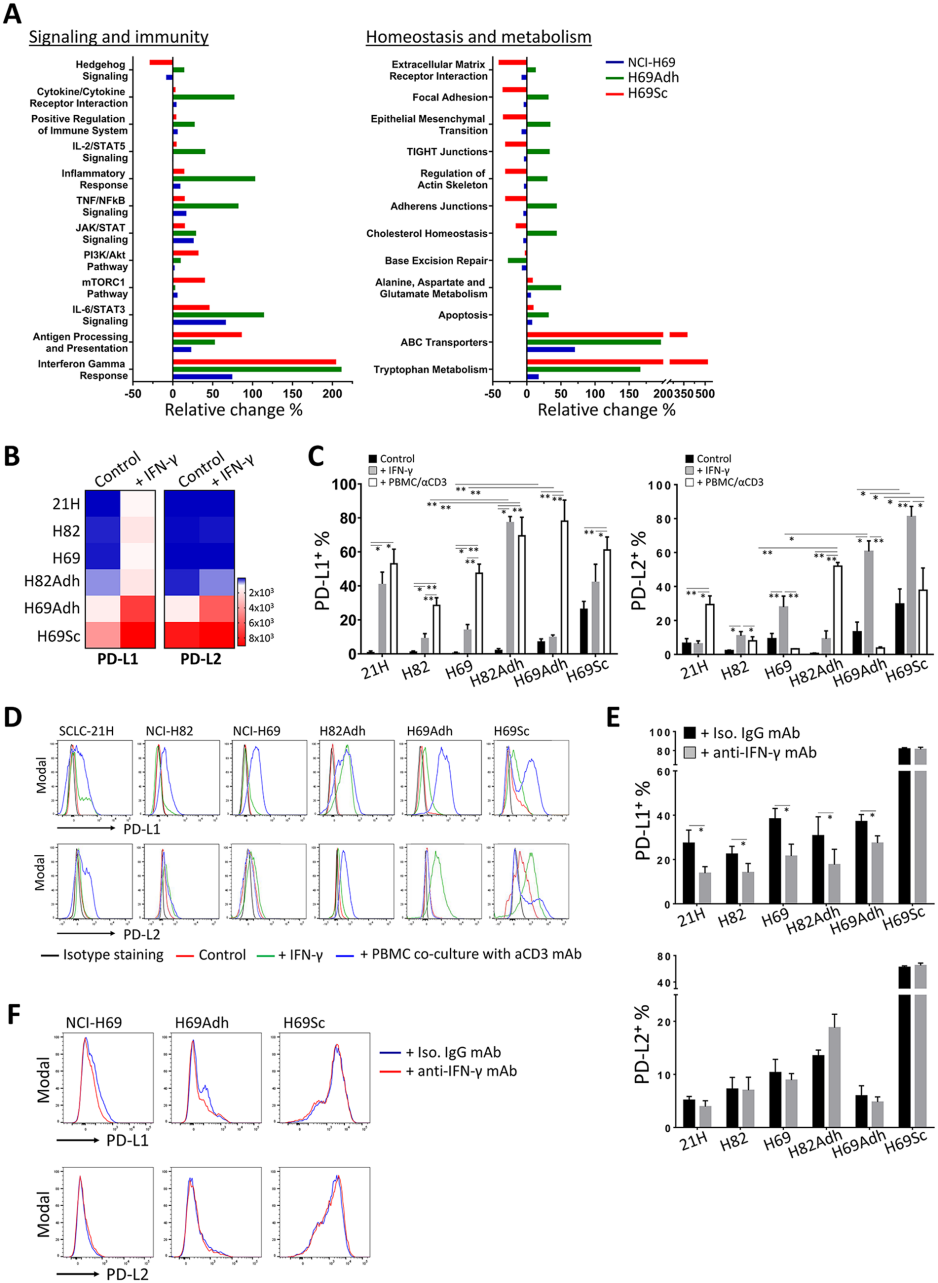
IFN-γ response and PD-1 ligand expression in SCLC subpopulations. **a** Percent enrichment analysis comparing the pathways modulated among NCI-H69 and its subpopulations after IFN-γ stimulation. **b** Relative change in the gene expression of PD-L1 and PD-L2 in the SCLC cells is presented as heatmaps. **c** Percentage distribution and **d** representative flow cytometry histograms of PD-L1^+^ and PD-L2^+^ SCLC cells under control conditions, after IFN-γ stimulation (150 ng/mL) for 48 h, or co-culturing with anti-CD3-stimulated PBMCs for 72 h. **e** The percentages and **f** representative flow cytometry histograms of PD-L1 and PD-L2 positivity on SCLC cells when co-cultured for 24 h with anti-CD3-primed PBMCs in the presence of IFN-γ blocking mAb or isotype-matched control mAb. (SCLC-21H, 21H; NCI-H82, H82; NCI-H69, H69; *n* ≥ 3, *p < 0.05, **p < 0.01)

Since IFN-γ was the most prominent cytokine produced in the co-cultures established with the SCLC cells and PBMC, next, the cells were treated with IFN-γ and the discrepancies between the parental SCLC cells and their adherent and stem-like derivatives were assessed. In response to IFN-γ, enhanced expression of the genes responsible for inflammatory responses and immune signaling pathways were noted especially in H69Adh cells and, to a lesser extent, in H69Sc cells (Fig. 3a). Correspondingly, IFN-γ response genes were explicitly upregulated in H69Adh and H69Sc cells, which indicated enhanced responsiveness and/or sensitivity of these cells to IFN-γ. Expression of the genes implicated in homeostatic and metabolic processes was generally augmented in H69Adh. In response to IFN-γ, the pathways related to cell adhesion and migration tend to decrease in H69Sc. Nevertheless, the highest expression levels of ATP-binding cassette (ABC) transporters and the genes involved in tryptophan metabolism were observed in the adherent H69 subpopulations, particularly in H96Sc (Fig. 3a).

Saliently, PD-L1 and PD-L2 were among the immune regulatory genes induced by IFN-γ. All SCLC lines used tended to increase the expression of PD-L1 upon exposure to IFN-γ. H69Adh and H69Sc highly upregulated both ligands at the mRNA level (Fig. 3b). Surface expression of PD-1 ligands, especially PD-L1, was augmented when the SCLC cells were either stimulated with IFN-γ or co-cultured with anti-CD3-activated PBMC (Fig. 3c, d). As observed at the gene expression level, the highest percentages of PD-L1^+^ and PD-L2^+^ cells were observed in the adherent populations of SCLC. Notably, in the absence of stimulation, a subpopulation of H69Sc cells constitutively expressed the PD-1 ligands (PD-L1^+^, 26.65 ± 4.23%; PD-L2^+^, 29.67 ± 8.87%) (Fig. 3c, d). In response to IFN-γ, CD44^+^ and/or CD90^+^ subpopulations were identified with higher levels of PD-L1 and PD-L2 (Supplementary Fig. 8). Moreover, neutralization of the IFN-γ secreted into the co-cultures by PBMC significantly hindered the upregulation of PD-L1, but not PD-L2, on SCLC cells. However, induction of PD-L1 upon co-culturing with PBMC was not alleviated on H69Sc cells by IFN- γ neutralization (Fig. 3e, f). Therefore, upon exposure to immune responses, the SCLC cells with mesenchymal and stem-like properties displayed enhanced capacities to upregulate co-inhibitory ligands, especially through IFN-γ-mediated pathways.

### Activated *T* cells become prone to checkpoint inhibition by mesenchymal and stem-like SCLC subpopulations

**Fig. 4:**
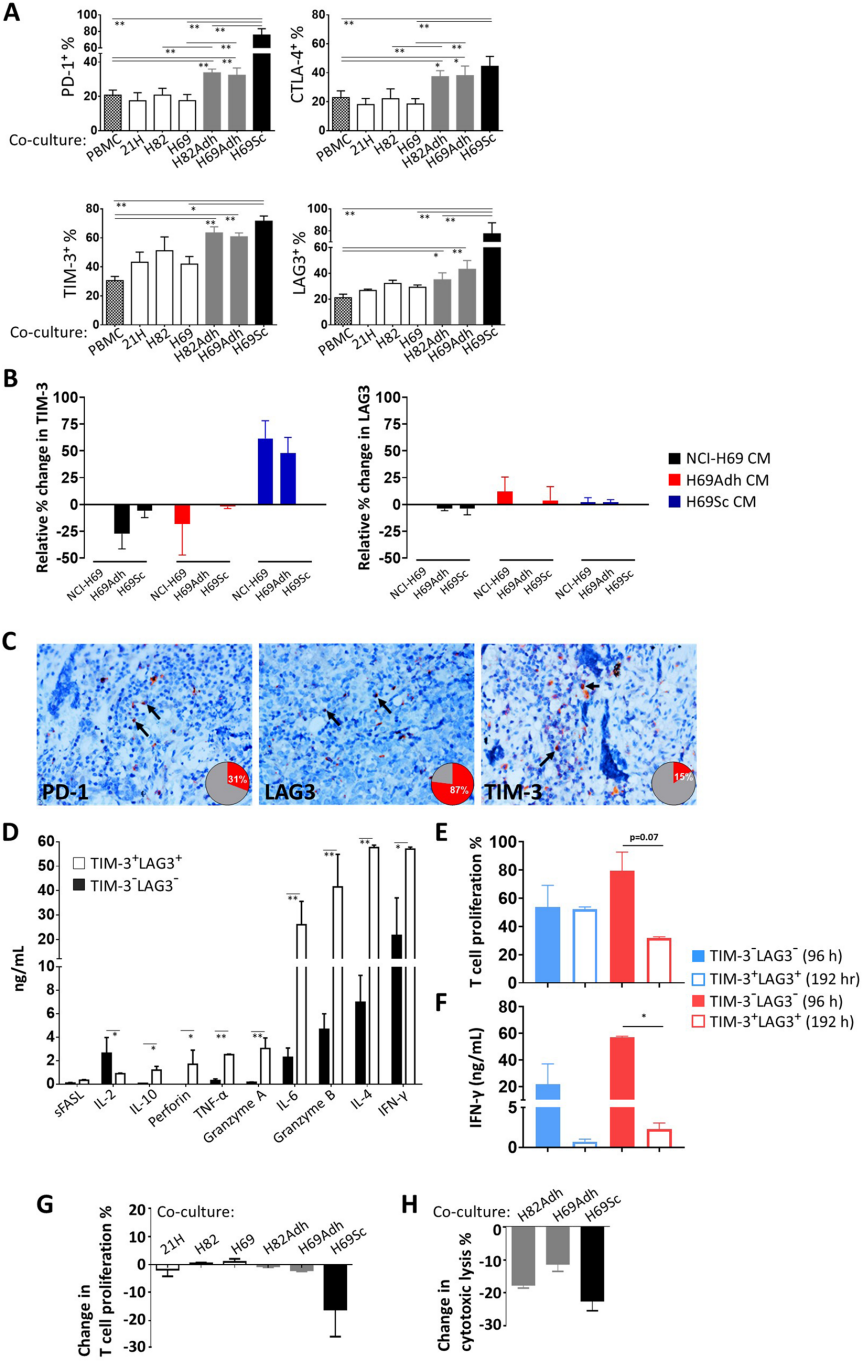
Expression of inhibitory receptors and the functional status of T cells co-cultured with SCLC cells. **a** Expression of PD-1, CTLA-4, TIM-3 and LAG3 on CD8^+^ T cells in the co-cultures of PBMCs and SCLC subpopulations. The cultures were established in the presence of anti-CD3 mAb (25 ng/mL) for 96 h at a 0.25:1 SCLC:PBMC ratio. **b** Relative expression change of TIM-3 and LAG3 on CD8^+^ T cells in the co-cultures of PBMCs and SCLC subpopulations treated with conditioned media collected from different subpopulations in 1:1 ratio. The cultures were established in the presence of anti-CD3 mAb (25 ng/mL) for 96 h at a 0.25:1 SCLC:PBMC ratio with CM collected after 48 h of cell culture. **c** Representative LAG3, PD-1 and TIM-3 immunohistochemical staining micrographs with checkpoint receptor expressing lymphocytes positive tumor-draining lymph node percentage in SCLC patients (magnification, 400x). TIM-3^−^LAG3^−^ and TIM-3^+^LAG3^+^ CD8^+^ T cells were purified from the 96 h or 192 h co-culturing of PBMCs and H69Sc cells, **d** cytokines produced were assessed by a flow cytometric bead array following 16 h stimulation with PMA and ionomycin upon 96 h, **e** proliferation capacity was assessed by CFSE dilution upon 72 h stimulation with anti-CD3 and anti-CD28 mAbs, **f** secreted IFN-γ amount with 16 h stimulation with PMA and ionomycin. CFSE-labelled PBMCs were co-cultured with IFN-γ-pretreated or control SCLC cells; the change **g** in CTL proliferation for 96 h and **h** CD3/CD28 dynabeads activated CTL co-cultured with IFN-γ-pretreated or control SCLC cells in SCLC cell lysis for 72 h was calculated. (PBMC, control PBMC alone; SCLC-21H, 21H; NCI-H82, H82; NCI-H69, H69; *n* ≥ 3, *p < 0.05, **p < 0.01)

Next, in the co-cultures of anti-CD3-stimulated PBMC and SCLC cells, wherein *T* cell responses were not directly suppressed, the expression of co-inhibitory receptors was assessed on CTLs. Following 96 h of co-culturing, the expression of PD-1, CTLA-4, TIM-3, and LAG3 was measured on CD8 + *T* cells (Fig. 4a). Especially when co-cultured with the adherent derivatives of NCI-H82 and NCI-H69 cells, the percentage of cells expressing these checkpoint receptors PD-1, CTLA-4, and LAG3 constituted at 30–40% of the CTLs. TIM-3 was upregulated by a larger fraction (> 60%) of CTLs. Intriguingly, the amount of PD-1^+^ (75.64 ± 7.69%), LAG3^+^ (77.35 ± 10.03%), and TIM-3^+^ (71.42 ± 3.63%) *T* cells was significantly increased when co-cultured with CD44^+^CD90^+^ H69Sc cells (Fig. 4a, Supplementary Fig. 7). In order to understand whether the induction of inhibitory receptor expression on CTLs was through soluble factors or a cell-contact dependent mechanism, conditioned media (CM) from different subpopulations were applied to co-cultures of NCI-69 cell line and its subpopulations (H69Adh and CD44^+^CD90^+^ H69Sc cells) with CTLs. Even though CM from CD44^+^CD90^+^ H69Sc cells induced TIM-3 expression on CTLs co-cultured with NCI-69 or H69Adh to some extent, LAG3 expression was not impacted by soluble factors (Fig. 4b). This finding evinced the need for cell-contact of stem-like SCLC cells to fully maintain their capacity to induce checkpoint receptor expression on CTLs. Correspondingly, positivity for these co-inhibitory receptors was also detected on the *T* cells infiltrating the metastatic foci in the SCLC patients’ lymph node samples, especially with common LAG3 positivity (Fig. 4c). Next, the CD8^+^ *T* cells (which were also positive for PD-1) were isolated from the 96 h co-cultures with H69Sc cells according to TIM-3 and LAG3 expression. The mediators related to effector functions of the expression profile of TIM-3^+^LAG3^+^ CTLs were significantly higher than that of the TIM-3^−^LAG3^−^ population (Fig. 4d). Accordingly, TIM-3^+^LAG3^+^ CTLs were able to more efficiently proliferate when isolated from the co-cultures (Fig. 4e). In conclusion, upon 96 h of co-culture with H69Sc cells, activated and TIM-3^+^ LAG-3^+^ expressing CTLs exert effector functions with clonal expansion and elevated pro-inflammatory cytokine secretion compared to TIM-3^−^ LAG-3^−^ CTLs possessing rather naïve-T cell characteristics (Fig. 4d, e, Supplementary Fig. 9). However, if the coculture is extended for a further 96 h, proliferation capacity of TIM-3^+^LAG3^+^ CTLs was significantly impaired by having even fewer proliferated cells than TIM-3^−^ LAG3^−^ cells upon anti-CD3/CD28 activation (Fig. 4e, Supplementary Fig. 9). Secretion of pro-inflammatory IFN-γ cytokine in response to a second activation after 192 h of H69Sc co-culture was hampered for both TIM-3^+^LAG3^+^ and TIM-3^−^LAG3^−^ CTLs (Fig. 4f). Strikingly, secretion of IFN-γ from TIM-3^+^LAG3^+^ CTLs decreased up to 2.26 ng/mL after 192 h of co-culture with H69Sc while the level was above 50 ng/mL when TIM-3^+^LAG3^+^ CD8^+^ T cells were stimulated after 96 h H69Sc co-culture. The alteration in the functional state of CTLs over time in SCLC co-culture with induction of multiple inhibitory receptor expression indicates T cell exhaustion and suppression in response to CSC subpopulation of SCLC.

In our experimental setting, since the inhibitory ligands, especially PD-L1 and PD-L2, were upregulated by SCLC cells following the IFN-γ-related immune reactions and the co-inhibitory receptors were upregulated on the CTLs; next, we sought if effector *T* cell responses are functionally hindered upon further interaction. For this purpose, the SCLC cells were pretreated with IFN-γ in order to induce immune regulatory pathways and co-cultures were again established with anti-CD3-activated PBMC or CD8^+^ *T* cells. In comparison with the PBMC cultured with control SCLC cells without IFN-γ pretreatment, IFN-γ-treated H69Sc cells reduced the CTL proliferation and cytolytic activity of CTLs approximately by 25% (Fig. 4g, h). Even though CTL proliferation was not modulated with other SCLC cells and their adherent derivatives, cytolysis of H82Adh and H69Adh cells was moderately reduced when these cells were treated with IFN-γ prior to co-culturing (Fig. 4g, h).

Collectively, upon interaction with the SCLC subpopulations bearing CSC-like and mesenchymal properties, the cytotoxic *T* cells can display effector functions including IFN-γ secretion and cytotoxicity; however, they also highly upregulate inhibitory checkpoint receptors and become prone to suppressive signals. Our data indicate the regulatory roles of CSC-like SCLC cells especially in the metastatic lymph nodes where SCLC cells inevitably interact with *T* cells.

## Discussion

SCLC demonstrates optimal first-line therapy responses; however, many patients succumb to the disease in a rather short period of time [4, 5]. The poor survival of SCLC patients is due to the tumor cells’ heterogeneity, the recurrence with inevitable drug resistance and distant metastasis capacity which are all linked to CSC in many other tumor types as well [11]. Candidate subpopulations with CSC features such as insensitivity to toxic materials, invasiveness, 3D growth and tumorigenicity at low numbers were previously reported in primary small cell lung tumors and cell lines. Adherence and expression of common stem cell markers CD44, CD90, CD87, and CD133 have been used for the identification of CSC [9, 33, 41–43]. We characterized the adherent subpopulations after serial passaging in NCI-H82 and NCI-H69 cell cultures. The adherent subpopulations showed greater surface area on fibronectin with significantly elevated directionality in H69Adh and induced tumor propagation capacity in nude mice. In terms of marker expression, common CD90 expression in SCLC cell lines and subpopulations suggested that CD90 is not a reliable CSC marker alone for SCLC. However, together with CD44 expression in adherent H69Sc, it augmented the migratory capacity of the cells and resembled the transcriptomic profile of MSC, especially in terms of the genes related to pluripotency, epithelial-to-mesenchymal transition, and migration. Here, CD44^+^CD90^+^ adherent SCLC cells were characterized and used as a prototype for CSC in SCLC. As MSCs possess the potential to promote metastasis by downregulating antitumor immune responses [35, 36], the presence of CD44^+^ SCLC cells was of abundance and in close contact with lymphocytes in the metastatic lymph nodes although they were scarce in primary tumors. Furthermore, the evidences such as the unique adaptive immune resistance mechanism emerged from CD44 expressing cells in squamous cell carcinoma (SCC) and positive correlation of CD44 expressing cell number with the tumor progression and relapse in SCLC support the potential role of CD44 expressing SCLC cells in tumor progression by developing adaptive resistance to immune destruction [23, 44]. Hence, characterization of immunomodulatory potential of CD44^+^CD90^+^ adherent SCLC cells offers a clinically relevant disease progression perspective in SCLC including a new candidate population for immunotherapy.

The immune system is programmed to discriminate and target non-self and stressed cells including tumor cells which possess altered-self antigen repertoire [45, 46]. SCLC is considered an immunogenic tumor type with its high mutation burden and common paraneoplastic syndromes observed in the patients [30, 31]. However, as also observed in our study, leukocyte and *T* lymphocyte infiltration are limited in primary SCLC lesions [40]. To our knowledge, immune modulation in the areas of metastasis is poorly understood; a dynamic model should be employed to better follow how *T* cell-mediated anti-tumor immune responses are regulated in the presence of SCLC cells, i.e. potentially CSC-like CD44^+^ fraction. By using a co-culture model, our study recapitulates the interaction between immune cells, especially CTLs, and the subpopulations of SCLC cells. Unlike many tumor cells of various tissue origins, SCLC cells did not directly suppress CTL responses. Compared to the leukocytes cultured alone, the in vitro milieu in the co-cultures even contributed to the augmentation of CTL-associated immune parameters. Nevertheless, the CSC-like subpopulations with mesenchymal properties showed the highest capacity to promote activation, proliferation, cytotoxicity and IFN-γ secretion. This intriguing situation has been previously implied by other studies performed on acute myeloid leukemia, basal-like breast cancer, and melanoma. As a common feature, these cancer types generally display conspicuous infiltration by *T* lymphocytes. This phenomenon has been described as an adaptive (secondary) immune resistance mechanism where the cancer cells start a suppression program via induction of regulatory molecule expression upon exposure to inflammatory mediators, such as IFN-γ [24, 25, 47].

With its cytostatic, cytotoxic, and immune-provoking effects, IFN-γ is one of the central mediators of anti-tumor immunity. As summarized before [26], three strategies are utilized by tumor cells to cope with the anti-tumor effects of IFN-γ: i) losing the sensitivity to IFN-γ, ii) shifting the signaling pathway from STAT1/IRF1 to rather pro-tumorigenic alternative pathways like STAT3/NF-κB, iii) up-regulation of inhibitory ligands such as PD-L1 and PD-L2. Only limited PD-L1 expression has been reported in SCLC cell lines and patient samples [48, 49]. Correspondingly, in non-small cell lung cancer (NSCLC), the absence of PD-L1 expression on tumor cells was associated with impaired IFN-γ response [50]. Considering the significant elevation of IFN-γ in the co-cultures of SCLC cells with activated immune cells, the transcriptome data and induction of PD-1 ligands indicate that particularly adherent or CSC-like subpopulations in SCLC respond to IFN-γ. Of note, these subpopulations are represented at very small numbers among (parental) SCLC cells; therefore, their impact on immune modulation is generally underscored.

Upon exposure to immune reactions which lead to the production of a plethora inflammatory mediators into the co-cultures, IFN-γ was determined the major factor that induced PD-L1, but not PD-L2. Apart from IFN-γ, numerous inflammatory factors were reported to have PD-L1 induction capacity including TNF-*α*, IL-1*α*, IL-17, IL-6, IL-8, EGF, IL-4, IL-27 and IL-10 [51, 52]. Similar to having various inducer factors, STAT1 is not the only transcription factor for PD-L1 expression. Pro-tumorigenic transcription factors such as Stat3, Myc, Kras that are also mediated with various inflammatory factors also play in role on PD-L1 expression [52]. Conversely, regulation of PD-L2 differed from PD-L1 by being regulated more through Th2 cytokines and Stat6 or NF-κB transcription factors if not induced through IFN-γ [53, 54]. Particularly, PD-L1 and PD-L2 expression in CD44^+^CD90^+^ CSC-like H69Sc cells were potentially sensitive to other inflammatory factors as well. This observation supports the notion that stem cells and CSCs are more adjustable to changing conditions and inflammation [55, 56]. Thus, SCLC cells and more explicitly CSC subpopulations show the signatures of adaptive resistance to better cope with anti-tumor immunity [24, 25].

Following activation, *T* cells upregulate inhibitory receptors that would hinder excessive responses and avoid immune-related pathologies [57, 58]. In the co-cultures, wherein the presence of SCLC cells did not impair CTL activation, inhibitory PD-1, CTLA-4, TIM-3, and LAG3 receptor expression were enhanced on the CTLs. This observation was in accordance with metastasis-infiltrating lymphocytes positive for PD-1, TIM-3, or LAG3. Therefore, in terms of PD-1 ligands, the upregulation of their cognate receptor would initiate a cycle of suppression. We did not test the expression of ligands for the inhibitory receptors other than PD-1; nevertheless, transcriptomic analyses indicated a negligible expression of the ligands for CTLA-4, TIM-3, and LAG3 on SCLC cells. Even though there was no evidence to claim the upregulated expression of ligands for alternative checkpoints such as TIM-3 and LAG3 on stem-like subpopulation of SCLC cells, the expression of these ligands such as Galectin-3 and Galectin-9 are regulated by inflammatory cytokines such as IFN-γ thus widely expressed on immune cells together with LAG3 ligand HLA-DR expression on monocytes/macrophages [59–61]. Hence, the presence of inhibitory ligands needs to be evaluated in the primary tumors and metastatic specimens of SCLC patients since expression of these ligands are also commonly found on stromal cells such as myeloid-originated immune cells.

High-level expression of multiple inhibitory receptors such as PD-1, LAG3 and TIM-3 on CTLs have been associated with a hyporesponsive state in which anti-tumor responses become ineffective. However, PD-1^+^LAG3^+^TIM-3^+^ CTLs were identified with potent effector functions, secretion of soluble mediators and proliferation. Therefore, CSC-like H69Sc cells did not induce T cell exhaustion which was examined as a further mechanism used by immunogenic tumors to hinder immunity. On the other hand, the functional status of CTLs infiltrating the lymph node metastases of SCLC must be further investigated.

**Fig. 5:**
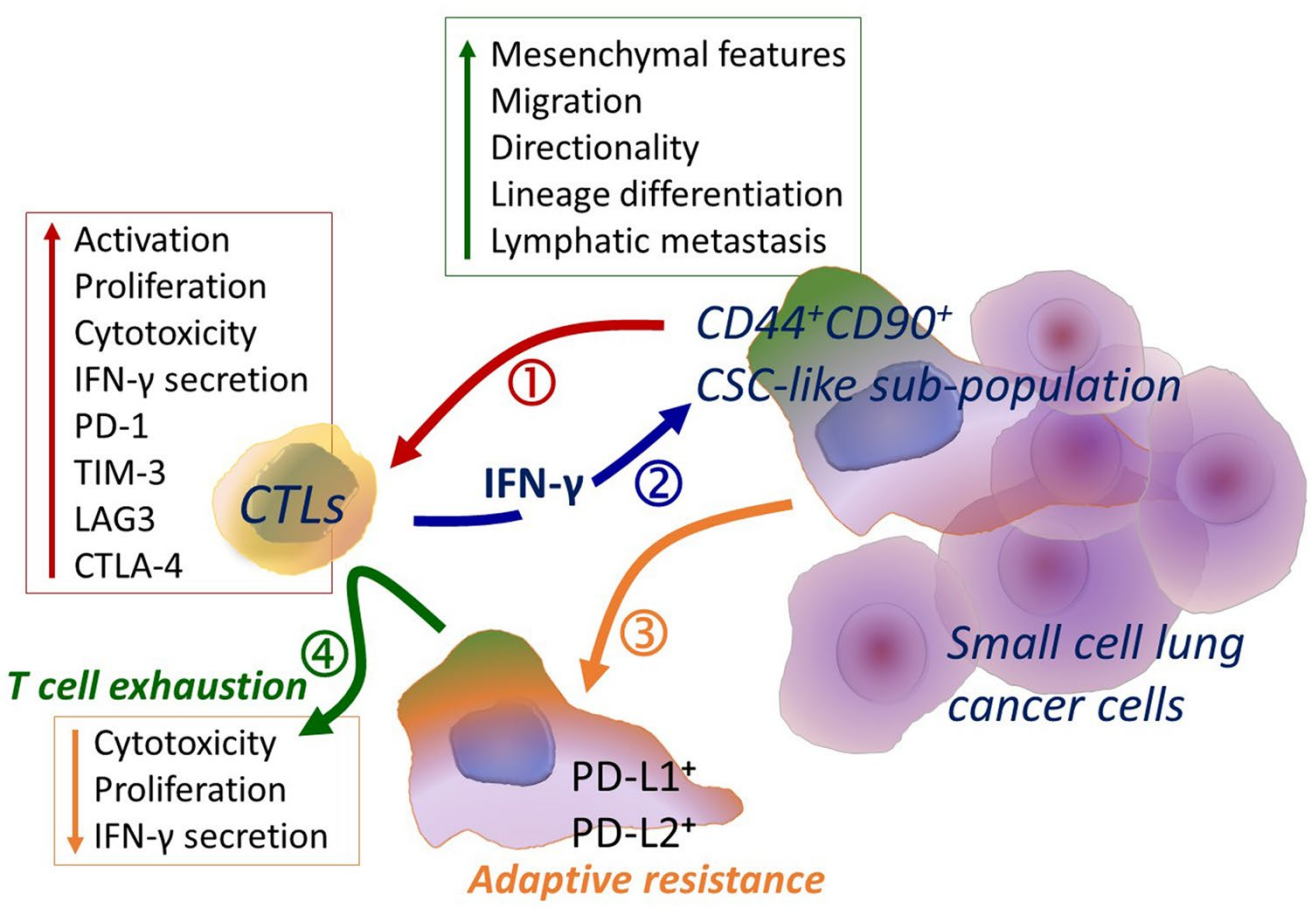
Schematic demonstration of the outcomes of CTL interaction with SCLC cells, primarily CSC-like subpopulation. CD44^+^ CD90^+^ CSC-like subpopulation of SCLC possessing mesenchymal features with elevated migration and lymphatic metastasis capacity (1) induces CTL activation, proliferation, cytotoxicity including high amount of IFN-γ secretion together with expression of checkpoint receptors on CTLs. (2) IFN-γ secreted by CTLs regulates phenotype, signaling and metabolism of the CSC-like subpopulation of SCLC cells with (3) augmented expression of PD-1 ligands indicating the adaptive resistance capacity of these cells. (4) Eventually, sustained inflammation and elevation of regulatory ligands in the environment result with T cell exhaustion that impairs proliferation capacity and cytotoxicity of CTLs

In conclusion, SCLC cells did not directly interfere with CTL responses and CSC-like adherent subpopulations, which are harbored by SCLC cells in very small numbers, can even provide appropriate milieu maintaining the *T* cell activation but induce the expression of inhibitory receptors. Thus, in their first encounter with SCLC cells, *T* cells may become activated, inducing their cytotoxic functions resulting in the death of SCLC cells. However, with chronic inflammation, our results determine a possible *T* cell exhaustion due to elevation of inhibitory receptor expression on CTLs together with possibly sustained activation signal and elevated inhibitory ligands in the environment (Fig. 5). Moreover, the adaptive resistance capacity of CSC-like SCLC cells in response to inflammatory mediators, especially IFN-γ, makes them less immunogenic through upregulation of PD-1 ligands (Fig. 5). It must be noted that, due to the immune exclusion in the primary tumor, this encounter is more likely to happen in metastatic sites including lymph nodes. An increased number of CD44^+^ SCLC found in advanced disease in addition to CD44 enrichment in metastatic lymph nodes support this theory. Even though PD-1/PD-L1 interaction inhibits *T* cell proliferation and killing to some extent, *T* cells become open for immune suppression with the common expression of multiple inhibitory receptors. In other words, targeting one checkpoint pathway might be overcome with the suppression signal from an alternative checkpoint receptor. This finding is consistent with the limited success of *α*-PD-1/PD-L1 therapies in SCLC patients and indicates a potential requirement of multiple targeting. Therefore, the CSC-like subpopulation in SCLC which correlates with metastatic potential and drug resistance may serve as a preferential target for combination checkpoint blockade immunotherapy.

## Conclusion

Cancer stem cells were identified as modulatory cells in acquiring therapy resistance and cancer progression in SCLC patients. Moreover, mesenchymal CSC-like SCLC cells displayed an immune-provoking impact on cytotoxic *T* lymphocytes which led to the upregulation of co-inhibitory receptors on CTLs thus *T* cell exhaustion upon prolonged activation. In response to CTL activation and IFN-γ secretion, CSC-like SCLC cells induced PD-L1 and PD-L2 expression to further limit the CTL responses. A better understanding of this immunomodulatory mechanism regulated by CSC-like cells might have an impact on novel cancer immunotherapy approaches for SCLC patients.

## Supplementary Information

Below is the link to the electronic supplementary material.

Supplementary file1 (PDF 1045 KB)
