# Supplementary material for: Small cell lung cancer stem cells display mesenchymal properties and exploit immune checkpoint pathways in activated cytotoxic T lymphocytes
Source: Cancer Immunol Immunother. 2021 Jul 6;71(2):445–59. doi: 10.1007/s00262-021-02998-1 (PMC8783896; doi:10.1007/s00262-021-02998-1)
Supplement: Supplementary file 1 — Supplementary file1 (PDF 1045 KB) [file 262_2021_2998_MOESM1_ESM.pdf]

Supplementary Figures

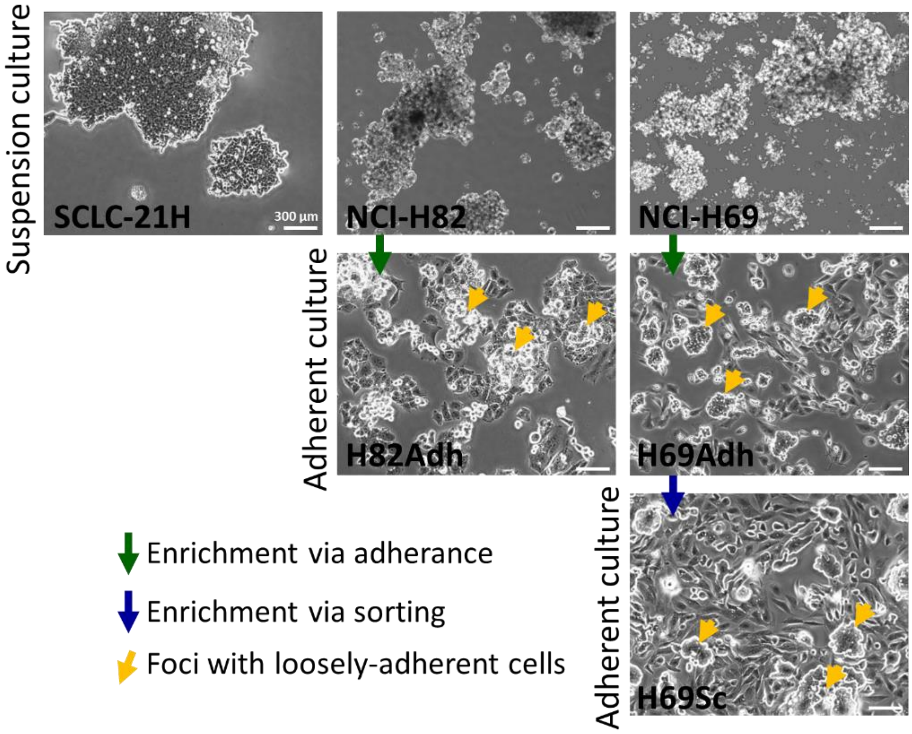

Supplementary figure 1. Representative inverted microscope images from (A) SCLC-21H, NCI-H82, NCI-H69 cells growing in suspension, (B) adherent subpopulations obtained as H82Adh and H69Adh, and (C) CD44^{+}CD90^{+} H69Adh cells are named as H69Sc.

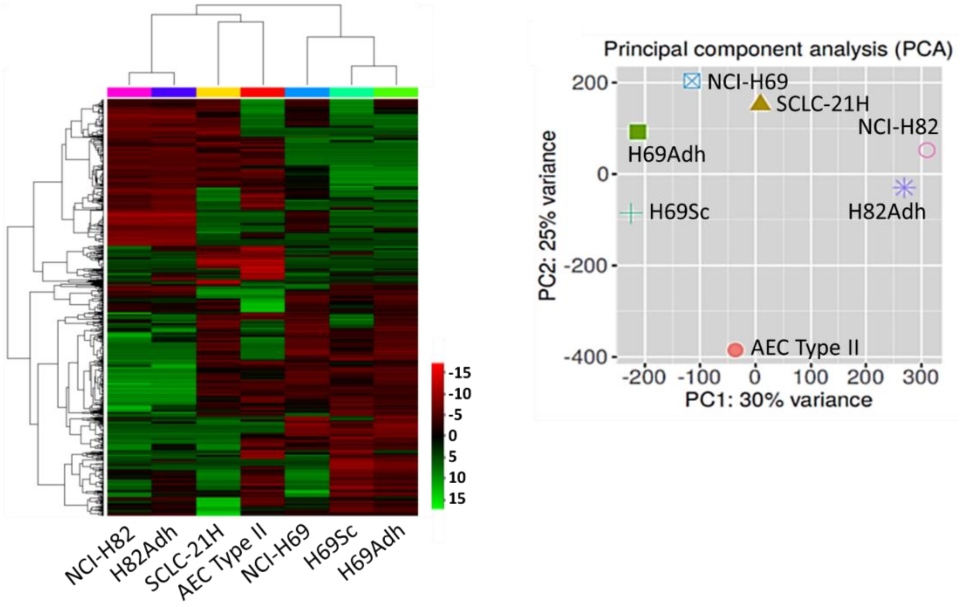

Supplementary figure 2. Global RNA expression heatmap analysis of SCLC cell lines and their subpopulations obtained are given in comparison with AECII cells. PCA distribution of the samples is shown in the right panel.

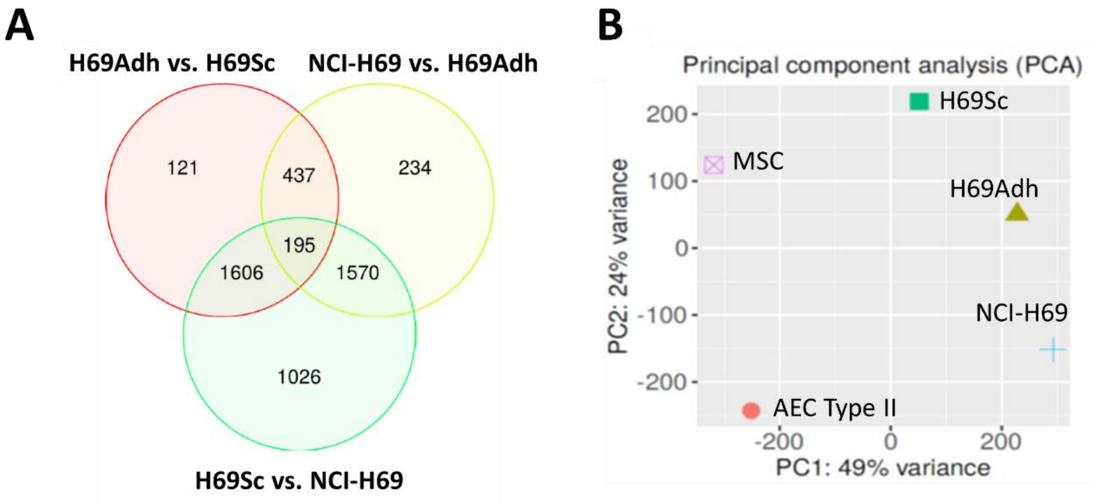

Supplementary figure 3. (A) Venn diagram of DEGs (8-fold difference) for NCI-H69 and its subpopulations H69Adh and H69Sc, **(B)** PCA distribution of the NCI-H69 and for NCI-H69 and its subpopulations are given in comparison with AECII cells and bone marrow-derived MSCs.

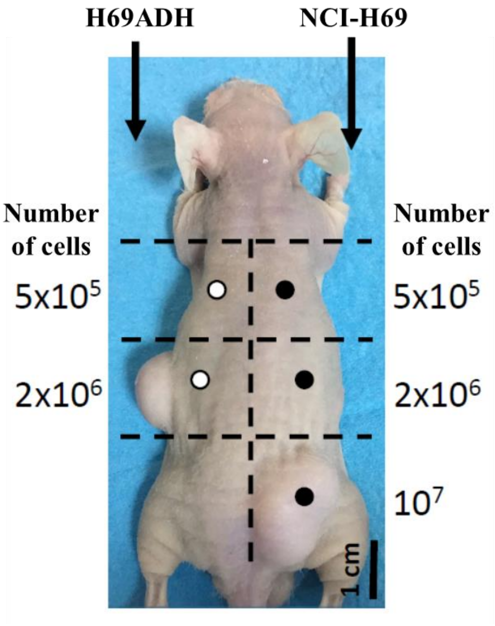

Supplementary figure 4. Tumor propagation capacity of NCI-H69 and H69Adh SCLC cells. A representative CD1-nude mouse was photographed after 4 weeks of subcutaneous inoculation of the tumor cells. Tumor growth was obtained with $2x10^6$ H69Adh cells whereas 107 NCI-H69 cells could form a tumor. The H69Adh cells were not inoculated at 10^7.

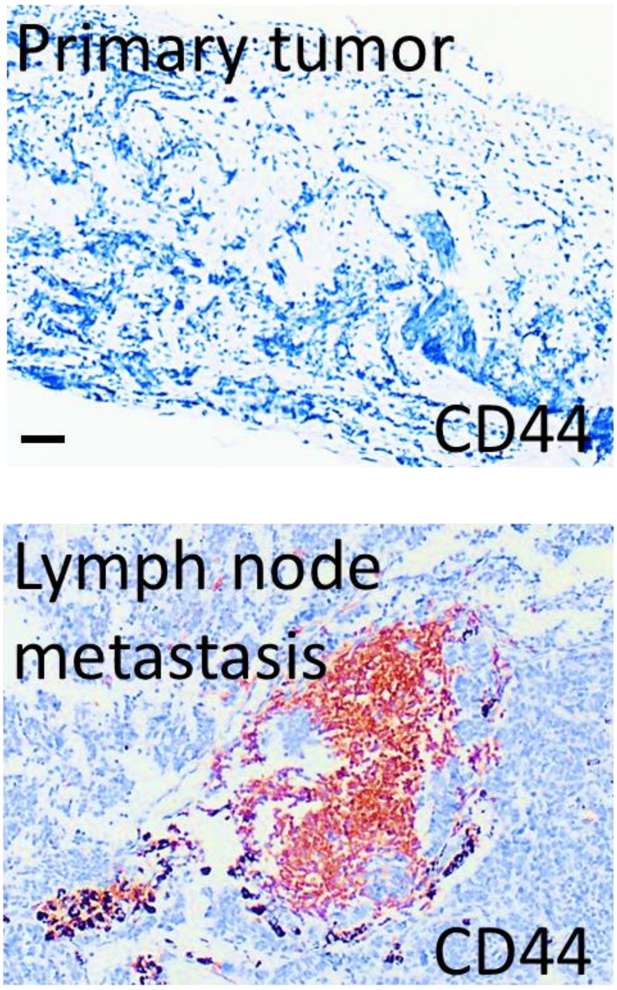

Supplementary figure 5. A representative micrograph of IHC staining for CD44 in a patient-derived primary SCLC tumor and a metastatic lymph node SCLC (magnification, 100x).

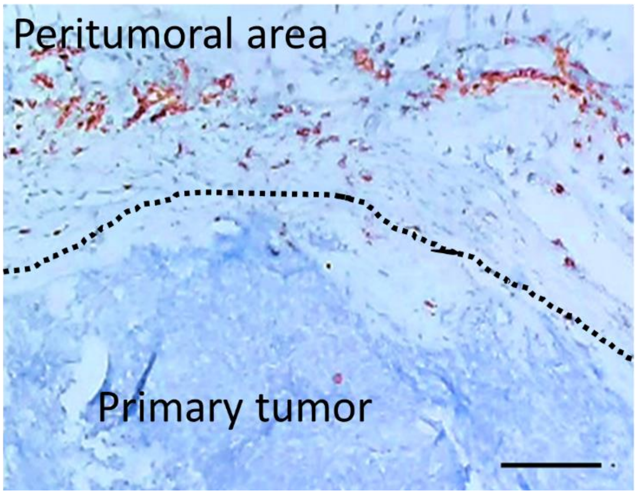

Supplementary figure 6. A representative micrograph of IHC staining for CD3 molecule indicating the peritumoral position of T cells in primary SCLC tumor specimens (Scale, 20x).

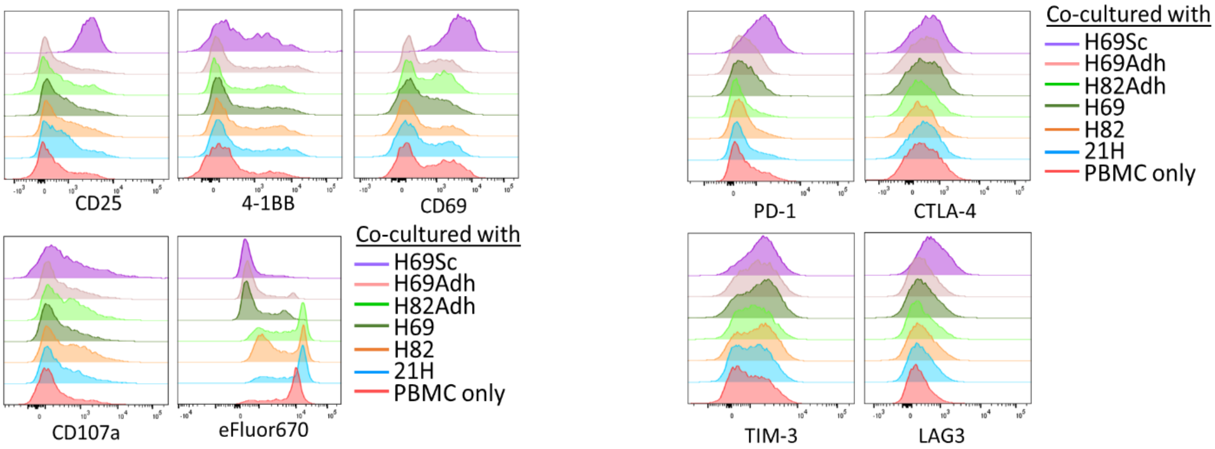

Supplementary figure 7. Representative overlay flow cytometry histograms of CD8$^+$ T cells gated amongst the PBMC co-cultured with SCLC cells are shown (0.25:1 SCLC:PBMC co-culture ratio in the presence of anti-CD3 mAb). CD25, 4-1BB, CD69, and CD107a data were obtained at 24 h whereas PD-1, CTLA-4, TIM-3, LAG3 expression and proliferation (eFluor670 dilution) were assessed at 96 h of co-culturing.

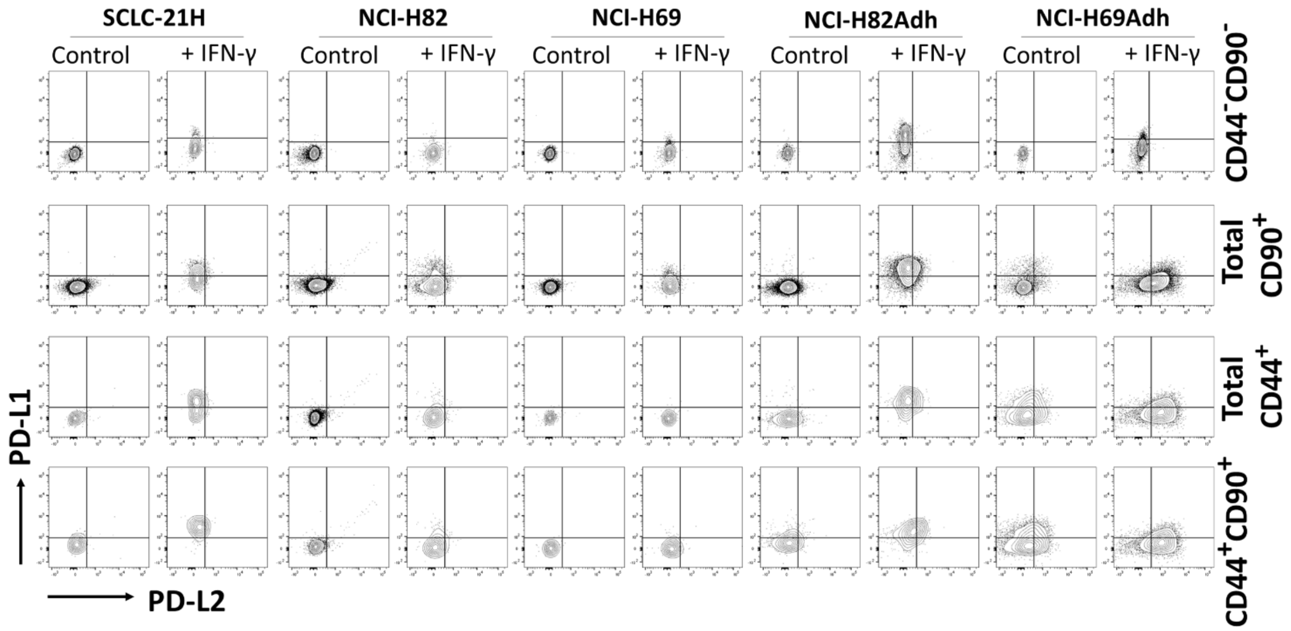

Supplementary figure 8. Representative counter plots of PD-L1 and PD-L2 expression on SCLC cells before and after 48h stimulation with IFN-γ (150 ng/mL). The cells were gated according to CD44 and CD90 expression.

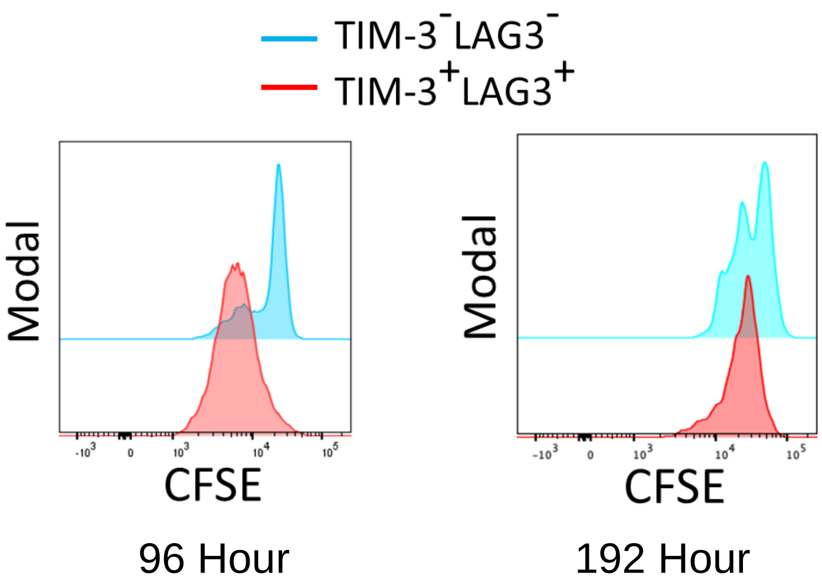

Supplementary figure 9. Representative CFSE dilution overlay flow cytometry histograms of TIM-3$^+$LAG3$^+$ CD8$^+$ T cells and TIM-3$^-$LAG3$^-$ CD8$^+$ T cells upon anti-CD3 and anti-CD28 mAbs. TIM-3$^+$LAG3$^+$ and TIM-3$^-$LAG3$^-$ CD8$^+$ T cells were sorted upon 96 hours of 192 hours of co-culture with H69Sc cells in 0.25:1 SCLC:PBMC co-culture ratio in the presence of anti-CD3 mAb.
